# *Major latex protein-like protein 43* (*MLP43*) functions as a positive regulator during abscisic acid responses and confers drought tolerance in *Arabidopsis thaliana*

**DOI:** 10.1093/jxb/erv477

**Published:** 2015-10-27

**Authors:** Yanping Wang, Li Yang, Xi Chen, Tiantian Ye, Bao Zhong, Ruijie Liu, Yan Wu, Zhulong Chan

**Affiliations:** ^1^Key Laboratory of Plant Germplasm Enhancement and Specialty Agriculture, Wuhan Botanical Garden, Chinese Academy of Sciences, Wuhan, Hubei Province, China; ^2^University of Chinese Academy of Sciences, Beijing, China; ^3^State Key Laboratory of Hybrid Rice, College of Life Science, Wuhan University, Wuhan, Hubei Province, China

**Keywords:** ABA signal reconstitution, abscisic acid, drought stress, metabolite profile, MLP43, reactive oxygen species, SnRK2.6.

## Abstract

Drought stress is one of the disadvantageous environmental conditions for plant growth and reproduction. Given the importance of abscisic acid (ABA) to plant growth and abiotic stress responses, identification of novel components involved in ABA signalling transduction is critical. In this study, we screened numerous *Arabidopsis thaliana* mutants by seed germination assay and identified a mutant *mlp43* (major latex protein-like 43) with decreased ABA sensitivity in seed germination. The *mlp43* mutant was sensitive to drought stress while the *MLP43*-overexpressed transgenic plants were drought tolerant. The tissue-specific expression pattern analysis showed that *MLP43* was predominantly expressed in cotyledons, primary roots and apical meristems, and a subcellular localization study indicated that *MLP43* was localized in the nucleus and cytoplasm. Physiological and biochemical analyses indicated that *MLP43* functioned as a positive regulator in ABA- and drought-stress responses in *Arabidopsis* through regulating water loss efficiency, electrolyte leakage, ROS levels, and as well as ABA-responsive gene expression. Moreover, metabolite profiling analysis indicated that *MLP43* could modulate the production of primary metabolites under drought stress conditions. Reconstitution of ABA signalling components in *Arabidopsis* protoplasts indicated that *MLP43* was involved in ABA signalling transduction and acted upstream of SnRK2s by directly interacting with SnRK2.6 and ABF1 in a yeast two-hybrid assay. Moreover, ABA and drought stress down-regulated *MLP43* expression as a negative feedback loop regulation to the performance of *MLP43* in ABA and drought stress responses. Therefore, this study provided new insights for interpretation of physiological and molecular mechanisms of *Arabidopsis MLP43* mediating ABA signalling transduction and drought stress responses.

## Highlight

MLP43 interacts with SnRK2.6 and ABF1 and functions as a positive regulator in ABA and drought stress responses through modulating gene expression, ROS production and primary metabolite profiles.

## Introduction

Abiotic stresses greatly affect plant growth and crop production. To date, plants have evolved many mechanisms to adapt and survive against these stresses, which include developmental, morphological, physiological and biochemical strategies. Plant hormones play essential roles in promoting and mediating these defence responses (Peleg and Blumwald, 2011). Abscisic acid (ABA) is regarded as a key signal involved in regulating the response of plants to various stresses, and particularly in regulating drought stress responses when plants experience water deficit (Cutler *et al.*, 2010; Lee and Luan, 2012). ABA production increases radically under drought stress conditions and stimulates stomatal closure, changing the expression of various osmotic stress-responsive genes (Kim *et al.*, 2010). In recent years, significant research progress has been made in studies using plants bearing gene mutations involved in hormone-biosynthetic and signalling transduction pathways, and to the identification of the ABA receptors PYR/PYL through chemical genetic approaches (Park *et al.*, 2009; Ma *et al.*, 2009; Hubbard *et al.*, 2010). Given the importance of ABA to plant physiology and development, identification of novel components involved in ABA signalling transduction is critical.

Major latex protein (MLP) was first identified from the latex of the opium poppy (*Papaver somniferum*) (Nessler *et al.*, 1990; Nessler and Burnett, 1992). The orthologues of *MLP*, called *MLP*-like proteins (*MLP*), were later found in *Arabidopsis*, soybean and tobacco (Aggelis *et al.*, 1997; Wu *et al.*, 2008). MLP proteins belong to the Bet v 1 family, also known as the pathogenesis related 10 (PR10)-like protein family, which contains proteins with low sequence similarity, but shares a similar three-dimensional structure (Radauer *et al.*, 2008). The Bet v 1 family is divided into 11 subfamilies, including MLP, PR10, cytokinin receptors and plant polyketide cyclase-like subfamilies (Radauer *et al.*, 2008). Most of the Bet v 1 proteins function through binding ligands, such as cytokinins, brassinolides and secondary metabolites and trigger downstream signal transduction (Koistinen *et al.*, 2005; Radauer *et al.*, 2008). In *Arabidopsis*, the ABA receptor RCAR/PYR/PYL family of START proteins share amino acid and structural similarity with Bet v 1 proteins (Ma *et al.*, 2009; Park *et al.*, 2009). Previous studies indicated that *MLPs* are down-regulated by blue light in the *cry1* loss-of-function mutant (Phee *et al.*, 2007). Two At*MLP*s (At2g01520 and At2g01530) could be induced by cis-cinnamic acid and promotes vegetative growth and delays flowering (Guo *et al.*, 2011). One report showed that *MLP*s were related to the translocation of hydrophobic compounds in the Cucurbitaceae family, which could contribute to decreasing the hydrophobic contamination of fruit (Inui *et al.*, 2013). Three *MLP*s, existing as duplicated gene pairs, were significantly down-regulated by oxidative stress through whole-genome DNA microarrays analysis, indicating that *MLP* might be involved in plants stress responses (Kim *et al.*, 2005). Interestingly, a study by Chen and Dai (2010) indicated that overexpression of *MLP* in *Arabidopsis* leads to salt stress insensitivity. The homologues of the *MLP* gene family are differentially expressed in various tissues; however, the molecular mechanism of MLPs in abiotic stress response is still elusive.

In this study, we screened numerous *Arabidopsis* T-DNA insertion mutants through seed germination assay, and identified the mutant *mlp43* with decreased ABA sensitivity in seed germination. The drought stress responses of the *mlp43* mutant and *MLP43*-overexpressed transgenic plants were also examined. The tissue-specific expression patterns and subcellular localization of *MLP43* were detected. Physiological and biochemical analysis indicated that *MLP43* was involved in ABA- and drought-stress responses in *Arabidopsis* through modulating primary metabolite profiling and gene expression. Reconstitution analysis of ABA signalling component assay in *Arabidopsis* protoplasts and direct interaction assay indicated that *MLP43* functions as a positive regulator in ABA signalling transduction and could directly interact with SnRK2.6 and ABF1 in yeast.

## Materials and methods

### Plant materials and growth conditions

*Arabidopsis thaliana* ecotype Columbia (Col-0) was used in this study and all transgenic plants were generated in a Col-0 background. The line of *mlp43* mutant (Salk_109337 and Salk_033347) was obtained from the Arabidopsis Biological Resource Center (ABRC; http://arabidopsis.org/abrc/) and identified by PCR. Primers used are listed in Supplementary Table S1 at *JXB* online. The mutants of *ost1-1* (Mustilli *et al.*, 2002), *aba1-1* (Koornneef *et al.*, 1982) and *abi1-1* (Koornneef *et al.*, 1984) are derived from the *Arabidopsis* accession Landsberg *erecta* (Ler). The *pyr1/pyl1/4* triple mutant (Park *et al.*, 2009) is derived from the *Arabidopsis* accession Col-0. Seeds were surface sterilized and sown on Murashige and Skoog (MS) agar plates containing full-strength MS salts, 0.8% (w/v) agar, and 1% (w/v) sucrose. Germination assay was performed with or without ABA on MS plates (Sigma, A1049). The seeds were stratified at 4ºC for 4 d in darkness and then transferred to growth chamber with 16h/8h light/dark cycle at 23ºC, or were directly sown in soil after stratification under the same conditions.

### Plasmids construction and transgenic plant generation

MLP43 cDNA was amplified using the primers MLP43-F and MLP43-R and cloned into Pro35S::GFP to generate Pro35S::MLP43-GFP fusion construct. The fused protein was then digested by XhoI/SacI and inserted into a binary vector pBA to generate pBA-MLP43-GFP construct. Promoter fragment of MLP43 (-1769bp before start code ATG) was amplified using primers ProMLP43-F and ProMLP43-R and subsequently cloned into the pBI101 to generate ProMLP43::GUS construct. The plasmids used in reconstituted ABA signaling pathway have been described by Lu *et al.* (2013). The detail information of plasmids used in yeast two hybrid (Y2H) assay is listed in Supplementary Table S2. The primers used for plasmids construction are listed in Supplementary Table S3. The mutated ABI5 was generated previously through site mutation (Wang *et al.*, 2013). All transgenic plants were generated by introducing *Agrobacterium tumefaciens* (strain GV3101) carrying the corresponding plasmids through floral dip-mediated infiltration into Col-0 background (Clough and Bent, 1998). Complementary transgenic plants of *Com-3* were generated by introducing a pBA-MLP43-GFP construct into *mlp43* mutant background.

### GUS histochemical analysis and subcellular localization

GUS signals were detected according to the method described by Jefferson *et al.* (1987). Plants were pretreated with or without 100 μM ABA for 3h, and then immersed in 90% acetone for 30min. After incubation in the GUS staining solution (0.5mg/ml X-Gluc, 50mM PBS, pH 7.0; 5.0mM potassium ferricyanide, 5.0mM potassium ferrocyanide, 0.1% Triton X-100) at 37ºC over night, the stained plants were washed with 70% ethanol overnight. Images were taken with an inverted microscope (SMZ1500, Nikon). For subcellular localization of *MLP43*, we transformed the plasmids into *Arabidopsis* rosette leaves (Col-0) by bombardment. Images were taken with an inverted microscope (TE2000U, Nikon) equipped with cool CCD (CoolSNAP HQ2, Roper Scientific). GFP fluorescence was acquired with 488nm excitation, and the chloroplast auto-fluorescence was acquired with 543nm excitation.

### Drought treatment, water loss analysis, and stomatal aperture measurement

For measurement of drought tolerance, water was withheld from 14-day-old plants comparable in size and growing in pots. After 21 d of drought treatment, the plants were re-watered. Survival rates were determined and the plants were photographed 2 d after re-watering. For measurement of water loss from detached leaves, the rosette leaves were detached from four-week-old plants and weighed at the indicated times. To analyse stomatal apertures, we incubated rosette leaves in a solution containing 50mM KCl, 10mM CaCl_2_ and 10mM MES (pH 6.15) for 3h under light condition. ABA was then added to the solution to a final concentration of 50 μM. Stomatal apertures were then measured after 30min and 1h of ABA treatment, respectively. For each experimental repeat, at least 50 stomata were counted and measured by photoshop software. The values of stomatal width to length ratios (V) were defined as: V≥0.5, open; 0.5 >V≥0.25, partially open; and V<0.25, closed.

### Comparison of EL, ROS contents and antioxidant enzymes activities

EL was determined from the detached aerial parts of drought-stressed plants with the indicated time points. The detailed procedure was performed as described by Wang *et al.* (2013). Superoxide radicals (O^−^) and hydrogen peroxide (H_2_O_2_) were detected by nitroblue tetrazolium chloride (NBT) staining and 3,3’-diaminobenzidine (DAB) staining, respectively, as described previously (Ramel *et al.*, 2009). Two-week-old seedlings were grown in soil, and then withheld water for the number of days indicated. Quantification of H_2_O_2_ content was determined using the method described by Hu *et al.* (2012). The activities of antioxidant enzymes were measured after drought treatment application followed by the procedure described by Wang *et al.* (2013).

### Gene expression analysis by real-time quantitative RT-PCR

Two-week-old plants were treated as indicated phytohormones or abiotic stresses. Total RNA was extracted using a plant RNA purification kit (Tiangen, Beijing, China). Equal amounts of RNA were used for reverse transcription with ReverTra Ace-α-TM (TOYOBO, Tokyo, Japan) according to the manufacturer’s instructions. The primers used in qRT-PCR were designed using web tool GenScript (https://www.genscript.com/ssl-bin/app/primer). The primers used for qRT-PCR experiment are listed in Supplementary Table S4.

### Metabolite profiling by gas chromatograph time-of-flight mass spectrometry

Plant samples for metabolite profiling were withheld water for two weeks from the 15th day after sowing in the growth chamber, and then all seedlings were harvested and immediately frozen in liquid nitrogen. The experimental procedure for extract preparation was performed as described previously (Lisec *et al.*, 2006). The total extracts were quantified by performing chromatography on GC-TOF MS (Agilent 7890A/5975C, USA). The detailed procedure has been described by Shi *et al.* (2014), with the data representing the mean values of three independent experimental repeats. The results were analysed using the Cluster 3.0 program (http://bonsai.hgc.jp/~mdehoon/software/cluster/) and visualized using Java Treeview (http://jtreeview.sourceforge.net/).

### Transient expression assay

Mesophyll protoplasts were isolated from four-week-old Col-0 plants according to the methods described previously (Yoo *et al.*, 2007). All of the plasmids used in this assay were prepared through purification with caesium chloride/ethidium bromide (Sambrook *et al.*, 1989). For the luciferase assay, protoplasts were harvested after 12-h incubation under light conditions at 23ºC with or without stimuli (50 μM ABA). The activities of LUC and GUS were measured with the GloMax-Multi Jr Single Tube Multimode Reader (Promega, USA). All experiments were repeated at least three times.

### Yeast two hybrid (Y2H) assay

The Y2H assay was carried out according to the instructions for the Matchmaker GAL4-based two-hybrid system (Clontech). The full-length sequence of *MLP43* was cloned and inserted into pGADT and pGBKT, respectively. Another two homologous genes, *MLP34* and *MLP168*, were cloned and inserted into pGBKT. We cloned *ABI5*, *SnRK2.2*, *SnRK2.3*, *PYL1/2/5/9/13* and *ABI1* and fused them into the pGADT vector, respectively. The genes of *SnRK2.6*, *SnRK2.8*, *SnRK2.10*, *PYR1*, *ABF1* and *ABF3* were cloned and fused into the pGBKT vector, respectively. The restriction sites and primer sequences for the plasmids used in this assay have been provided in Supplementary Tables S2 and S3, respectively.

## Results

### The mlp43 mutant was insensitive to ABA during seed germination

**Fig. 1.**
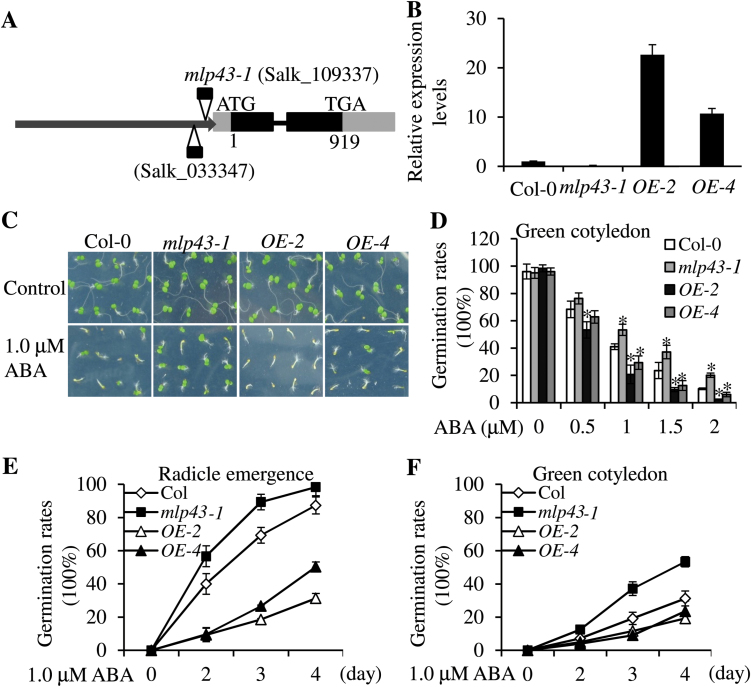
*MLP43* was involved in ABA responses in seed germination assay. Asterisk symbols (*) indicate *P*<0.05 (Student’s *t*-test). (A) Diagram showing of the T-DNA insertion site of *mlp43-1* mutant. (B) Relative expression levels of *MLP43* in the *mlp43-1* mutant and overexpressed transgenic plants. Expression levels of *β-ACTIN8* represent the internal control. Data represent the means ±SEs of three replicated experiments. (C) Seeds growing on MS medium with or without 1.0 μM ABA for 5 d after stratification. Both overexpressed transgenic plants (*OE-2* and *OE-4*) showed a hypersensitive response to ABA. Photographs were taken to document the phenotypes. (D) Germination rates of green cotyledons with the indicated ABA application. Germination rates (%) were scored 5 d after stratification. Data represent means ±SEs of three replicated experiments (*n*>60 for each experiment). (E) Germination rates of radicle emergence in 1.0 μM ABA plates at the indicated time points. Data represent the means ±SEs of three replicated experiments (*n*>60 for each experiment). (F) Germination rates of green cotyledons in 1.0 μM ABA plates at the indicated time points. Data represent the means ±SEs of three replicated experiments (*n*>60 for each experiment). (This figure is available in colour at *JXB* online.)

To discover other novel regulators and to expand ABA signalling networks during seed germination and abiotic drought stresses, we screened various T-DNA insertion mutants purchased from the ABRC (http://www.arabidopsis.org/) on MS medium containing 1.0 μM ABA during seed germination. The mutant *mlp43-1* (Salk_109337) showing insensitivity to ABA was selected for further anlysis. T-DNA was inserted into the 5’-UTR of *MLP43* (Fig. 1A). Transgenic plants of overexpressed *MLP43* (*OE-2* and *OE-4*) was generated by introducing Pro35S::MLP43-GFP plasmids into Col-0 plants. The relative expression level of *MLP43* was verified by qRT-PCR (Fig. 1B). The expression levels of MLP43-GFP fusion protein was detected using anti-GFP antibody through western-blot assay (Supplementary Fig. S1A). In the absence of ABA, no obvious differences were observed in germination rates between Col-0 and the *mlp43-1* mutant (Fig. 1C, D). However, the *mlp43* mutant showed significantly decreased ABA sensitivity on the 1.0 μM ABA plate, while overexpressed *MLP43* enhanced ABA sensitivity during seed germination (Fig. 1C, D). Moreover, the kinetics of germination time of Col-0, *mlp43-1*, and *MLP43 OE* were compared in the presence of 1.0 μM ABA by analysing the percentages of emerged radicles and open green cotyledons (Fig. 1E, F). On the 4th day after stratification, about 80% and 25% of Col-0 emerged with radicles and green cotyledons, respectively. However, the corresponding percentages of *mlp43-1* were more than 95% and 50%. Overexpressed *MLP43* transgenic plants showed much lower germination rates than Col-0 plants (Fig. 1E, F). We also examined the ABA sensitivity with another T-DNA insertion Salk line *mlp43-2* (Salk_033347) in the seed germination assay. As indicated in Supplementary Fig. S1, this mutant line harbours almost null expression levels of *MLP43* and was insensitive to ABA in seed germination. The ABA sensitivity of root growth was also examined after ABA treatment and the results indicated that no significant differences in primary root growth and lateral root number were observed among Col-0, *mlp43-1*, and *MLP43 OE* seedlings (Supplementary Fig. S2A, B). Taken together, *MLP43* might function as a positive regulator in ABA response during seed germination.

### Modulation of MLP43 expression by ABA and abiotic stress treatments

**Fig. 2.**
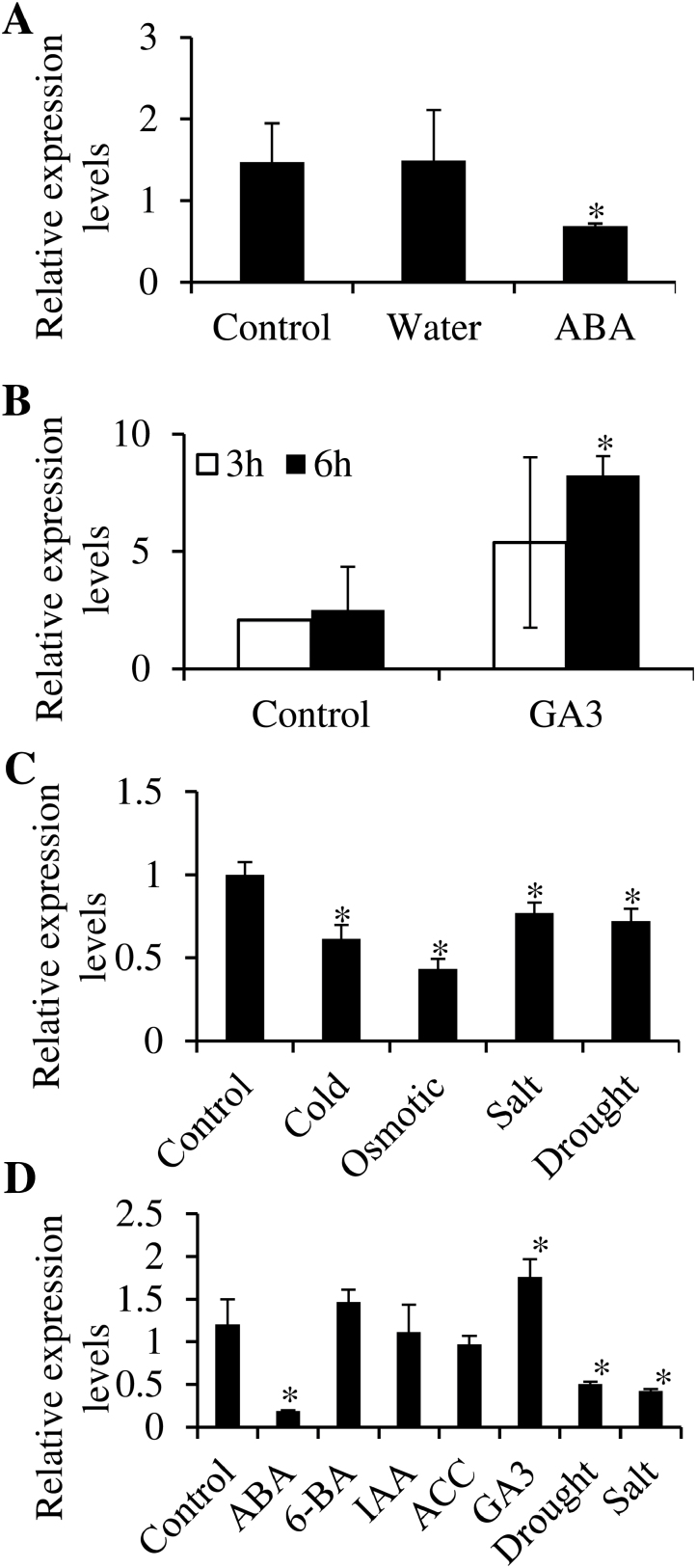
ABA and abiotic stresses affected *MLP43* expression. Data represent the means ±SEs of three replicated experiments. Asterisk symbols (*) indicate *P*<0.05 (Student’s *t*-test). (A) Publicly available microarray data illustrating the expression level of *MLP43* was inhibited by 30 μM ABA treatment for 24h (http://www.bar.utoronto.ca/NASCArrays/index.php?ExpID=183), but was (B) induced by gibberellic acid 3 in seeds (GA_3_, 5 μM, treated for different time courses) (http://www.bar.utoronto.ca/NASCArrays/index.php?ExpID=184). (C) Publicly available microarray data illustrating the effect of abiotic stress treatment (cold, osmotic, salt and drought) on expression level of *MLP43 (http://bar.utoronto.ca/efp/cgi-bin/efpWeb.cgi).* (D) Effects of hormones and abiotic stresses on *MLP43* expression using qRT-PCR. ABA (10 μM, 3h), 6-BA (1 μM, 3h), IAA (5 μM, 3h), ACC (5 μM, 3h), GA_3_ (10 μM, 3h), drought (water withheld for two weeks), salt (NaCl, 100mM, 3h). The values of gene expression levels of Col-0 seedlings without any treatment were taken as ‘1’. Expression levels of *β-ACTIN8* represent the internal control.

Based on publicly available microarray data (http://bar.utoronto.ca/efp/cgi-bin/efpWeb.cgi; Winter *et al.*, 2007), the *MLP43* transcript was inhibited by ABA (30 μM) after 24h of treatment in seeds, but induced by gibberellic acid 3 (GA_3_, 5 μM) after 3 and 6h of treatment, respectively (Fig. 2A, B). The effects of abiotic stresses on *MLP43* transcripts were further analysed. Interestingly, cold (4ºC), osmotic stress (300mM d-mannitol), salt (150mM NaCl) and drought (air dry) negatively regulated *MLP43* expression (Fig. 2C). To further confirm the effects of phytohormones and abiotic stresses on *MLP43* expression, qRT-PCR was applied to examine relative expression levels of *MLP43*. The results indicated that *MLP43* was actually inhibited by ABA (50 μM, 3h), drought (water withheld for two weeks) and salt (NaCl, 100mM for 3h) treatment, respectively. No significant changes were observed after cytokinin (6-BA), auxin (IAA) or ethylene (ACC) treatment except that GA_3_ treatment slightly up-regulated *MLP43* expression (Fig. 2D). These results showed that ABA and drought treatment decreased *MLP43* transcription, which were consistent with the public microarray data.

### Overexpressed MLP43 confers enhanced drought tolerance

**Fig. 3.**
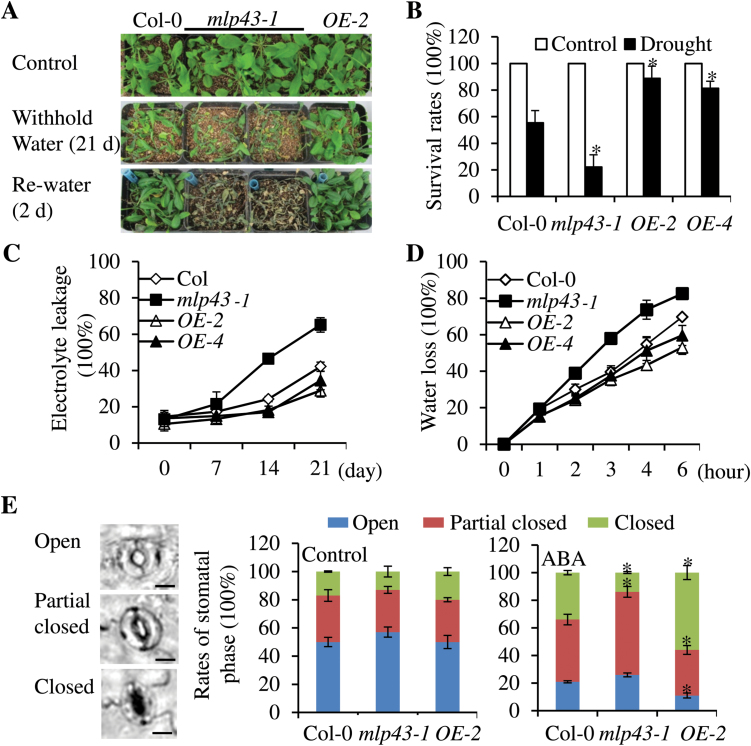
*MLP43* involvement in drought stress responses. (A) Two-week-old plants were well watered (control) or deprived of water for 21 d and then rewatered. The photos were taken two days after rewatering. (B) Survival rates after the drought treatment in panel A. Data represent the means ±SEs of three replicated experiments (*n*>20 for each genotype, *P*<0.05). (C) Electrolyte leakage comparison at the indicated time points after drought treatment applied. Data represent the means ±SEs of three replicated experiments (*n*=15 for each genotype). (D) Transpirational water loss from well-watered detached leaves of four-week-old plants. Water loss rates are indicated as the percentage of the initial fresh weight (% FW). Results are shown as the means ±SEs of three replicated experiments (*n*=15 for each genotype). (E) Stomatal aperture under ABA treatment. The stomata phases are defined as shown in left panel. Scale bar, 5 μm. The rates of different stomata phases are calculated with or without ABA (30 μM) treatment for 30min. The data represent the means of four replicated experiments (*n*>50, *P*<0.05). (This figure is available in colour at *JXB* online.)

As the *mlp43* mutant showed significantly decreased ABA sensitivity in seed germination, and drought treatment inhibited *MLP43* expression (Figs 1, 2), the responses to drought stress of the *mlp43-1* mutant and *MLP43*-overexpressed plants were further examined. Two-week-old plants grown under normal conditions were withheld water for three weeks, and then rewatered for 2 d. The results indicated that only 20% of *mlp43-1* mutants but more than 80% of *MLP43 OE* plants recovered from wilting after rehydration, while the survival rate of Col-0 was about 58% (Fig. 3A, B). The EL test showed that the *mlp43-1* mutant exhibited a significantly higher EL percentage than Col-0, but *MLP43 OE* plants showed a lower EL percentage relative to Col-0 (Fig. 3C). Transpirational water loss from detached leaves of 4-week-old plants were further compared at room temperature with a humidity of ~50–60%. Much higher water loss rates were detected in the *mlp43-1* mutant compared with Col-0, while *MLP43 OE* plants showed lower water loss rates compared to Col-0 (Fig. 3D).

ABA-induced stomatal closure is usually responsible for plant adaptation to drought stress. We defined the stomatal phases as open, partially open and closed, according to the ratios of width to length of stomatal aperture (Fig. 3E). Under normal conditions, the rates of different stomatal phases were comparable among wildtype (Col-0), *mlp43-1* and *MLP43 OE*. However, after ABA treatment for 1h, the rates of closed stomata were significantly increased in Col-0, especially in *MLP43 OE*. In contrast, the *mlp43-1* mutant exhibited fewer closed stomata and more partially closed stomata than those in Col-0 and *MLP43 OE* (Fig. 3E). Together, these results suggested that the improved drought tolerance of *MLP43 OE* was associated with an increased sensitivity to ABA-induced stomatal closure in *MLP43 OE*. The complementary overexpressed transgenic plants generated by introducing Pro35S::MLP43-GFP into *mlp43-1* were further assessed for drought resistance. The results indicated that the hypersensitivity of *mlp43-1* mutant to drought stress could be restored by *MLP43* overexpression (Supplementary Fig. S3).

### Tissue-specific expression patterns and subcellular localization of MLP43

**Fig. 4.**
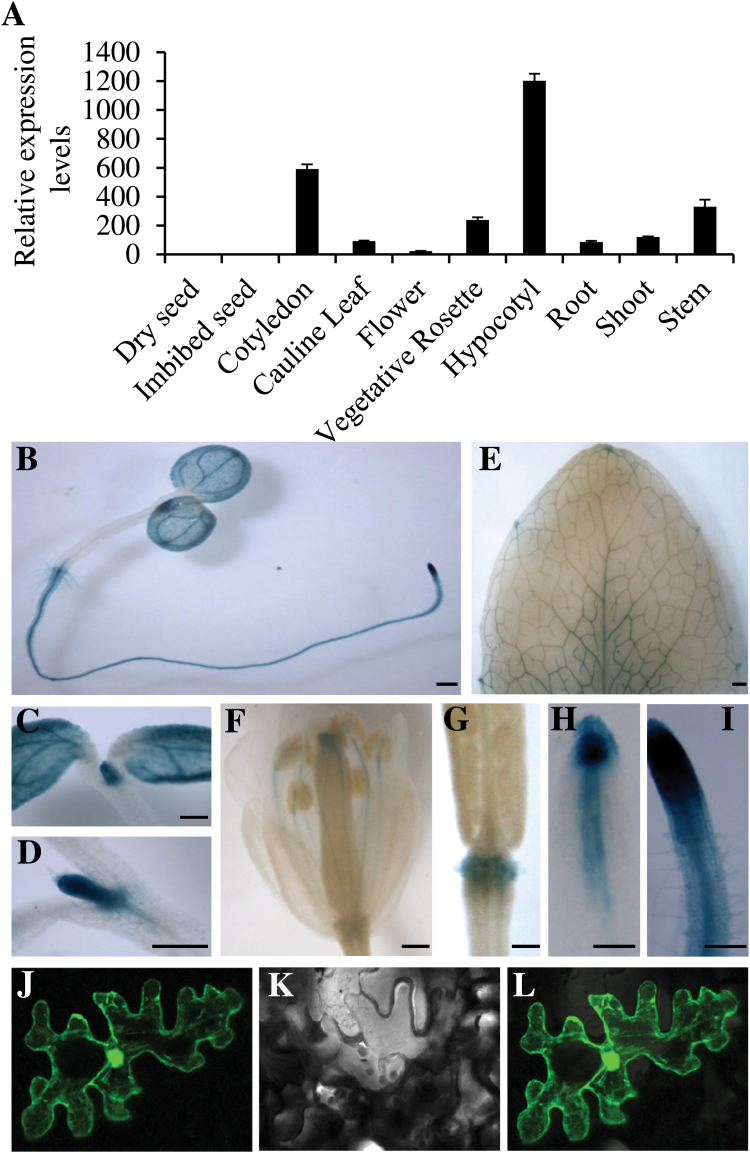
Tissue-specific expression patterns of *MLP43*. (A) Publicly available microarray data illustrating the tissue specific expression levels of *MLP43 (http://bar.utoronto.ca/efp/cgi-bin/efpWeb.cgi).* (B)–(I) Analysis of tissue-specific expression patterns by GUS histochemical staining. Scale bar, 0.5mm. (J)–(L) Subcellular localization of MLP43-GFP fusion protein drived by the 35S promoter. Bar, 10 μM. Photos taken under cofocal microscope J, GFP; K, bright field; L, overlay. (This figure is available in colour at *JXB* online.)

Tissue-specific expression patterns of *MLP43* were firstly analysed based on the microarray data available in public resources (Fig. 4A; http://bar.utoronto.ca/efp/cgi-bin/efpWeb.cgi; Winter *et al.*, 2007). The results indicated that *MLP43* preferentially expressed in cotyledon and hypocotyls in *Arabidopsis,* while lower expression levels could be detected in roots, shoots, stems and vegetative rosette leaves (Fig. 4A). Almost no expression patterns could be detected either in dry seed or in imbibed seeds (Fig. 4A). To further confirm the specific expression patterns of *MLP43* in *Arabidopsis*, the promoter sequence of *MLP43* (−1769 bps upstream of start code ATG) was cloned and a transgenic plant carrying plasmid ProMLP43::GUS was generated. GUS histochemical signals were analysed in various tissues (Fig. 4B–I). There were predominant GUS signals in cotyledons, primary roots and apical meristems (Fig. 4B–D, H, I). GUS signals also could be detected in rosette leaves, flowers and the abscission zone (Fig. 4E–G) whereas no GUS signals had been examined in hypocotyls, which was inconsistent with the microarray data. We transiently expressed the Pro35S::MLP43-GFP fusion construct into epidermal cells of *Arabidopsis* rosette leaves by bombardment and observed the subcellular localization of the MLP43-GFP fusion protein under a confocal fluorescence microscope. The results indicated that MLP43 were localized in nucleus and cytoplasm in *Arabidopsis* epidermal cells (Fig. 4J–L).

### Mutation of MLP43 increased ROS production and modulated primary metabolites after drought treatment

**Fig. 5.**
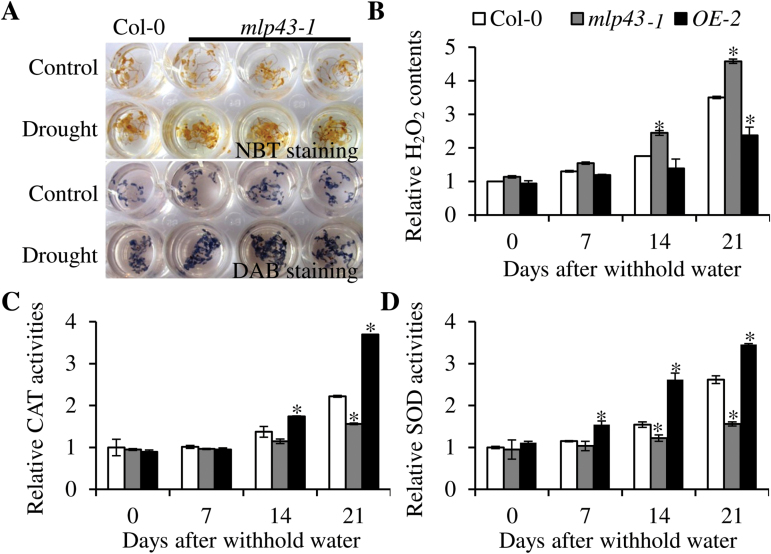
*MLP43* enhanced drought stress tolerance through inhibiting ROS production and promoting antioxidant enzyme activities. (A) Visualization of superoxide radical and hydrogen peroxide detected by NBT and DAB staining using two-week-old MS-grown plants subjected to subsequent treatment with or without air dry for 30min. Comparison of (B) H_2_O_2_ content, (C) CAT and (D) SOD activity in Col-0, *mlp43* mutant and overexpressed transgenic plant *MLP43 OE*. Two-week-old plants were well watered and then withheld water for the indicated number of days. The relative contents of H_2_O_2_ and the activity of CAT and SOD were calculated as fold-changes compared with the Col-0 control. Data represent the means ±SEs of three replicated experiments (*n*=10; *P*<0.01). (This figure is available in colour at *JXB* online.)

ABA and drought stress trigger ROS accumulation, and a ROS detoxification mechanism is necessary to enable plant survival under drought stress (Smirnoff, 1993). To characterize whether ROS accumulation was altered in the *mlp43-1* mutant, we determined H_2_O_2_ and O^−^ production by DAB and NBT staining, respectively. The *mlp43-1* mutant exhibited a dark colour relative to Col-0 with both DAB and NBT staining after air drought stress treatment, indicating more accumulation of H_2_O_2_ and O^−^ in the *mlp43-1* mutant (Fig. 5A). We also measured the H_2_O_2_ content in the young seedlings, and found that drought stress induced rapid accumulation of H_2_O_2_ after 14 d of drought treatment in all genotypes. However, the H_2_O_2_ level was significantly higher in the *mlp43-1* mutant plants, but lower in *MLP43 OE* when compared with Col-0 plants (Fig. 5B). Next, the enzymatic activities of SOD and CAT were measured under the same drought conditions. The results indicated that no significant differences of CAT activity were observed among three genotypes after drought treatment for 7 d, however, SOD activities were 1.5-fold higher in *MLP43 OE* plants than wildtype (Col-0) and the *mlp43-1* mutant, respectively (Fig. 5C, D). Moreover, both SOD and CAT activities showed a significant decrease in *mlp43-1* and increase in *MLP43 OE* relative to Col-0 plants after drought treatment for 14 d and 21 d, respectively.

**Fig. 6.**
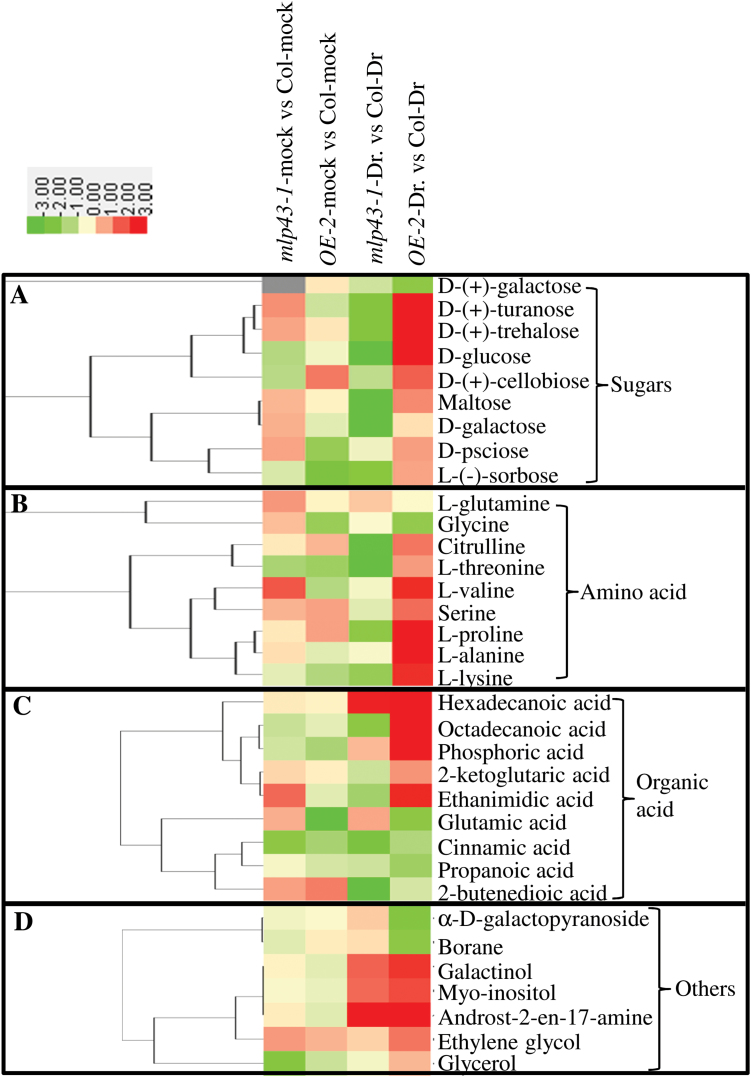
Comparisons of metabolite profiling under control and drought treatment in Col-0, *mlp43-1* and *MLP43 OE* plants. Thirty-four compounds that could reproducibly be detected by GC-TOF MS in all three genotypic plants were analyzed by hierarchical clustering. The resulting tree was analyzed using CLUSTER 3.0 and shown using Java Treeview. (This figure is available in colour at *JXB* online.)

To elucidate the metabolic responses of *MLP43* to drought stress treatment, GC-TOF-MS was applied to test whether the primary metabolite profiling was modified by *MLP43* overexpression. The results indicated that 34 metabolites including 9 carbohydrates, 9 amino acids, 9 organic acids and 7 other derivatives were reproducibly identified (Fig. 6; Supplementary Table S5). Compared with wildtype (Col-0), most of the detected metabolites showed <2-fold differences in *MLP43* ectopic expression lines without drought treatment. However, the contents of most of the examined carbohydrates and amino acids were significantly decreased in the *mlp43-1* mutant, but increased in *MLP43 OE* transgenic plants after drought treatment (Fig. 6). Except D-(+)-galactose, all carbohydrates, including maltose, sorbose, cellobiose, turanose, glucose, psicose and galactose, decreased in the *mlp43-1* mutant but increased in *MLP43 OE* when compared to Col-0 under the withheld water condition (Fig. 6). A similar change was observed for citrulline, threonine, valine, serine, proline, alanine and lysine, with the exception of glutamine and glycine (Fig. 6). In addition, hexadecanoic acid, galactinol, myo-inositol and androst-2-en-17-amine increased, while cinnamic acid, propanoic acid and 2-butenedioic acid decreased either in *mlp43-1* mutants or in *MLP43* transgenic plants under drought treatment conditions.

### Transcriptional alterations of ABA- and drought-responsive genes by MLP43

**Fig. 7.**
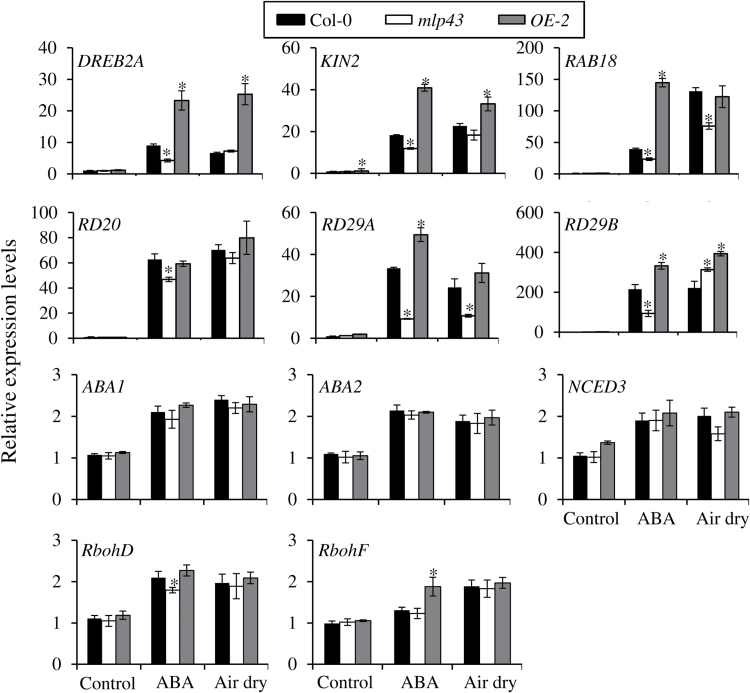
Relative expression levels of drought- and ABA-responsive genes in *mlp43-1* mutant and *MLP43 OE* plants. The two-week-old plants were subjected to ABA treatment (50 μM) for 3h and air dried for 6h, respectively. The expression levels of the selected drought- and ABA-responsive genes were analysed quantitatively by qRT-PCR. Gene expression levels were analysed relative to the corresponding gene expression levels in Col-0 plants. The values of gene expression levels of Col-0 seedlings without ABA treatment were taken as ‘1’. Expression levels of *β-ACTIN8* represent the internal control. The data represent means ±SEs of three reproducible experiments (*P*<0.01).

To further determine the role of *MLP43* in the ABA signalling pathway and drought stress responses, we assessed the expression patterns of genes involved in ABA- and drought-responsive processes. Two-week-old seedlings were pretreated with 50 μM ABA for 3h or air dried for 6h, respectively, after which gene expression levels were analysed by qRT-PCR. We compared the expression levels of several ABA- and/or abiotic stress-responsive genes, including *DREB2A*, *KIN2*, *RAB18*, *RD20*, *RD29A* and *RD29B*. All of them showed extensively increased transcriptional levels after ABA and air-dry treatments in all three genotypes (Fig. 7). However, the fold changes of *DREB2A*, *KIN2*, *RAB18* and *RD29A* were significantly lower in the *mlp43-1* mutant, but higher in the *MLP43 OE* plants compared to those in Col-0. ABA treatment significantly enhanced the transcription of above ABA- and drought-responsive genes in *MLP43 OE* plants, which was consistent with the ABA hypersensitivity of *MLP43 OE* in seed germination and stomatal aperture responses. Dehydration stress usually promotes ABA production and actives ABA signal transduction (Chan, 2012), and our results substantiate this, with ABA and air-dry treatments inducing similar expression patterns of the above detected genes (Fig. 6). In addition, we also examined expression level changes of three ABA biosynthesis genes, *ABA1*, *ABA2* and *NCED3*. The results indicated that there were no significant differences among the three genotypes after ABA or air-dry treatments. As MLP43 modulated ROS production, we also examined the relative expression levels of *RbohD* and *RbohE* encoding NADPH oxidases which promote ROS production especially after ABA and drought treatment (Torres *et al.*, 2002; Miller *et al.*, 2010). Results revealed no significant differences except that *RbohE* showed a relatively higher expression level in the presence of ABA (Fig. 7, last panel). Based on the above results, we concluded that the overexpression of *MLP43* enhanced plant drought stress adaptation through an ABA-dependent manner.

### MLP43 positively modulated ABA responses in reconstituted ABA signalling pathway

**Fig. 8.**
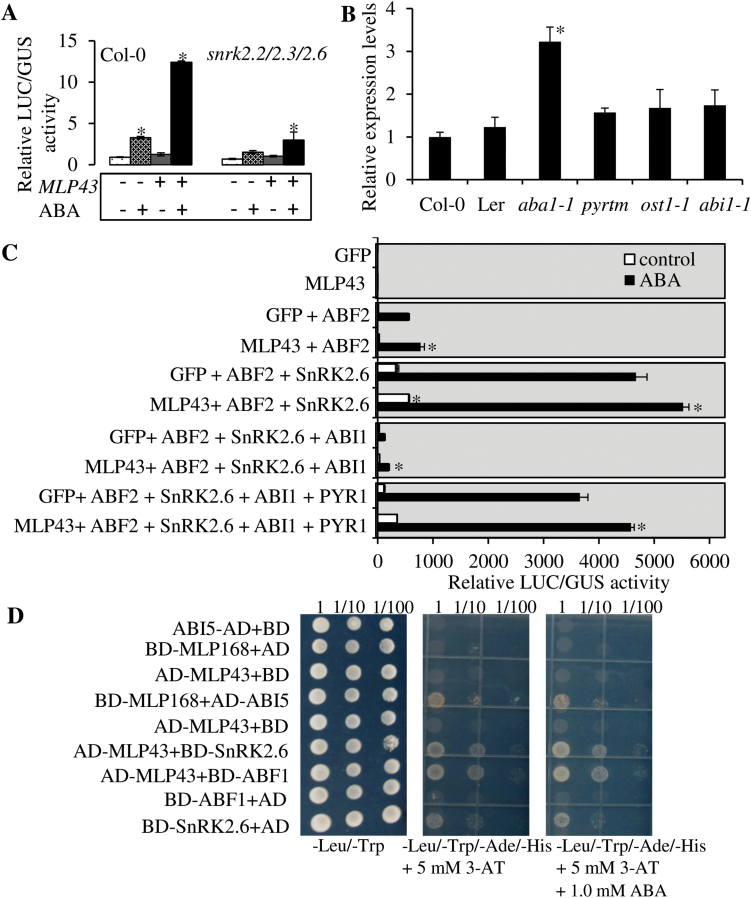
*MLP43* functioned as a positive regulator in the reconstituted ABA signalling pathway. The data represent means ±SEs of five reproducible experiments (*P*<0.01). (A) Relative activities (LUC/GUS) in co-transformation with empty vector (Pro35S::GFP) or MLP43 (Pro35S::MLP43-GFP) in Col-0 and *snrk2.2/2.3/2.6* with or without ABA (50 μM) treatment for 12h, respectively. (B) Relative expression levels of *MLP43* in the primary ABA signal components related gene mutants using qRT-PCR. The value of gene expression levels of Col-0 seedlings was taken as ‘1’. Expression levels of *β-ACTIN8* represent the internal control. (C) Relative activities (LUC/GUS) in the reconstituted ABA signal pathway. Co-transformation of *MLP43* enhanced the relative activities (LUC/GUS). ABF2, SnRK2.6, ABI1 and PYR1 were co-transformed as effectors, respectively. (D) Interactions between MLP43 and SnRK2.6 or ABF1 using the yeast two-hybrid assay. The homologous gene MLP168 could interact with wildtype ABI5 in yeast.

To elucidate how *MLP43* modulated the expression of downstream targets of the ABA signalling pathway, we performed a transient expression assay in *Arabidopsis* mesophyll protoplasts extracted from Col-0 and *snrk2.2/2.3/2.6*, respectively, using ProRD29A::LUC as reporter and ProUBQ::GUS as internal control, and Pro35S::GFP as empty vector control. The results indicated that transient overexpression of *MLP43* significantly enhanced the transcriptional activity of RD29A promoter after ABA treatment in Col-0 but not in *snrk2.2/2.3/2.6* (Fig. 8A). We then checked expression patterns of *MLP43* in mutants of primary ABA signalling components, including the *pyr1/pyl1/pyl4* triple mutant *(pyrtm*), *ost1-1*, *abi1-1* and the ABA-deficient mutant *aba1-1*. When compared with wildtype (Col-0 and Ler), the expression level of *MLP43* was up-regulated in all the examined mutants, especially in the ABA-deficient mutant *aba1-1* with ~3-fold increases at the transcriptional level (Fig. 8B). In order to gain more clues about the effect of *MLP43* in the ABA signalling pathway, we reconstituted the ABA signalling pathway in wildtype (Col-0) protoplasts (Fig. 8C). As expected, co-transformation of ABF2 and SnRK2.6 could extensively activate RD29A::LUC activity and PYR1 inhibited ABI1 activity and then enabled expression of the ABA-dependent transcription of RD29A::LUC (Fig. 8C). However, in the presence of *MLP43* overexpression, the activity of RD29A::LUC significantly increased, especially after ABA treatment when compared with empty vector control (Fig. 8C).

We further examined the interactions between *MLP43* and the key ABA signal components. First, the phylogenetic relationships among nine *MLP* homologous genes were analysed based on the full length amino acid sequences (Supplementary Fig. S4). Another two *MLP*s (*MLP34* and *MLP168*), which shared differencial homology with *MLP43*, were selected for the yeast two-hybrid assay together with *MLP43*. The results indicated that MLP43 could interact with SnRK2.6 and ABF1 in the yeast two-hybrid assay independent of ABA (Fig. 8D; Supplementary Fig. S5). However, we did not detect any interactions between MLP34 or MLP168 and the key ABA signal components, except the interaction between MLP168 and wildtype ABI5 protein (Supplementary Fig. S5A). Interestingly, mutated proteins carrying single or triple phosphoamino acid mutations (S42A, S145A and T201A) in ABI5 could eliminate its interactions with MLP168 (Supplementary Fig. S5A). Taken together, the above results indicated that *MLP43* might function as a positive regulator through interacting with SnRK2.6 and ABFs in ABA signalling responses, and ABA could negatively regulate the expression of *MLP43* as a complementary mechanism to modulate the function of *MLP43 in vivo*.

## Discussion

In this study, the function of MLP43 was characterized during plant stress responses. Both MLP43 and ABA receptors PYL/PYR belong to the Bet v 1 family and share similar protein structure. We speculated that *MLP43* might be involved in the ABA signalling pathway. The efficiency of water loss is responsible for plant tolerance to drought stress, and rapid water loss leads to greater drought sensitivity. As indicated, the rosette leaf water loss of the *mlp43-1* mutant was significantly more rapid than wildtype (Col-0) under the same drought stress conditions, resulting in higher electrolyte leakage and lower survival rates (Fig. 3). The above results were consistent with ABA insensitivity of *mlp43* in seed germination. As we know, ABA and GA_3_ antagonistically regulate seed germination (Lee *et al.*, 2002; Piskurewicz *et al.*, 2008). Interestingly, *MLP43* expression was significantly inhibited by ABA, but promoted by GA_3_ (Fig. 2A). Moreover, drought and salt treatments also negatively regulated *MLP43* expression. We also analysed the 1769bp promoter sequence of *MLP43* and found two ACGT-containing ABRE-like elements in the promoter region, located at −1291bp to −1294bp and −1088bp to −1092bp, respectively. Interestingly, a functional G-box (CACGTG) was also identified in the *MLP43* promoter, located at −603bp to −608bp close to the ATG start codon of *MLP43*. The occurrence of both an ABRE and G-box motif in the *MLP43* promoter is consistent with previous studies that indicated that ABRE/G-box elements were usually co-recognized by ABA responsive transcription factors (Menkens *et al.*, 1995; Shen and Ho, 1995; Ho *et al.*, 1999).

To further elucidate the mechanism of *MLP43* in mediating ABA and drought responses, the comparisons of expression levels of ABA- and drought-responsive genes were performed. As expected, the expression levels of *DREB2A*, *KIN2*, *RAB18*, *RD29A* and *RD29B* were up-regulated by ABA treatment, but showed lower expression levels in *mlp43-1* and higher expression levels in *MLP43 OE* when compared with those in Col-0 after ABA treatment. These results indicated that *MLP43* enhanced ABA signal transduction and drought stress tolerance through modulating downstream targets of ABA and drought stress responses. In addition, expression levels of ABA biosynthesis-related genes, such as *ABA1*, *ABA2* and *NCED3* showed no significant changes in *mlp43* and *MLP43* OE lines indicating that *MLP43* modulated ABA responses independent of the ABA biosynthesis pathway. However, *MLP43* expression was up-regulated in the ABA receptor triple mutant *pyr1/ pyl1/pyl4*, as well as in *ost1-1*, *abi1-1* (dominant negative mutant) and *aba1-1* mutants, when compared with those in wildtype Col-0 and Ler (Fig. 8B). In other words, disturbance of ABA signalling transduction or ABA biosynthesis blocked the inhibitory effect of ABA on *MLP43* transcription, indicating that the above key ABA signal components are important inter-mediators for the negative regulation of ABA on *MLP43* expression. Moreover, the re-constituted ABA signal pathway confirmed that transient over-expression of *MLP43* in protoplasts enhanced ABA responses by determining the reporter RD29A::LUC activity (Fig. 8C). However, mutation in *SnRK2.2/2.3/2.6* eliminated the positive effect of *MLP43* on ABA responses in the transient expression assay, which provides evidence that *MLP43* functions up-stream of *SnRK2*s in the ABA signal pathway (Fig. 8A).

Furthermore, the interactions between MLP43 and SnRK2.6 or ABFs could be detected in the yeast two-hybrid assay (Fig. 8D; Supplementary Fig. S5), which provided direct evidence for the involvement of MLP43 in the ABA signalling pathway. In the core ABA signalling pathway, PYL/PYRs interact with PP2Cs in the present of ABA to form PYL/PYR-PP2C complexes, which in turn inhibits the activity of the PP2Cs in an ABA-dependent manner, allowing activation of SnRK2s (Ma *et al.*, 2009; Park *et al.*, 2009). To date, no direct interactions between ABA receptors PYL/PYRs and SnRK2s have been reported. Therefore, even though MLPs and the ABA receptor RCAR/PYR/PYL family of START proteins share amino acid and structural similarity at the protein level, MLP43 does not interact with ABI1, a key negative regulator of PP2Cs in the ABA signalling pathway. We speculated that MLPs might function as positive ABA regulators through direct regulation of downstream SnRK2s or ABF activities. Solid evidence through the yeast two-hybrid assay verified that MLP43 interacts with SnRK2.6 and ABF1 (Fig. 8D). However, the MLP homologous genes might be functionally diverse in their ABA responses as there were no interactions between MLP34/MLP168 and other ABA signalling components, except that MLP168 could interact with ABI5 in yeast (Supplementary Fig. 5). Presence or absence of ABA had no different effect on the interactions between MLPs and SnRK2/ABFs, indicating the interaction between MLP43 and SnRK2.6 or ABF1 is ABA independent. Therefore, our study provides experimental evidence to elucidate how *MLP43* functions as a positive regulator in ABA signalling and drought stress responses.

Accumulating evidence indicates that ABA-enhanced water stress tolerance is associated with induction of antioxidant defence systems, including ROS-scavenging enzymes such as SOD, CAT and APX (Mittler *et al.*, 2011). ROS are small molecules generated during development and in response to stress, and function as eukaryotic intracellular second messengers (Finkel, 1998; Mittler *et al.*, 2011). In *Arabidopsis*, *AtrbohD* and *AtrbohE* encode NADPH oxidases which catalyse NADPH into NADP and ROS especially after ABA and drought treatment (Torres *et al.*, 2002; Kwak *et al.*, 2003; Miller *et al.*, 2010). Our results showed that mutation of *MLP43* increased ROS accumulation under drought stress conditions (Fig. 5A), indicating the involvement of *MLP43* in ROS-mediated drought responses. However, *MLP43* did not modulate *RbohD* and *RbohF* transcript levels according to the qRT-PCR results, which indicated that *MLP43* preferably regulated ROS scavenging but not production (Fig. 7). The further accumulation of excessive ROS causes more severe oxidative damage to plant cells and stimulates antioxidase activities (Mittler, 2002). SOD and CAT are two important antioxidases that scavenge excessive ROS to prevent cell damage. We determined significant increases of both SOD and CAT activities in *MLP43*-overexpressed transgenic plants (Fig. 5C, D), which suggests that *MLP43* played a role in scavenging the excessive ROS production under dehydration to enhance drought tolerance.

During plant abiotic stress responses, compatible solutes are important in helping plants balance external osmotic pressure and maintain cellular function of macromolecules. In this study, we further examined primary metabolic profiles using GC-TOF MS. The results indicated several carbohydrates and amino acids accumulated in *MLP43* plants (Fig. 6). The increased amounts of alanine and glutamic acid in *MLP43* OE plants during drought stress treatment might regulate photosynthesis intensity (Bocian *et al.*, 2015). Moreover, overexpression of *MLP43* resulted in accumulation of free proline, maltose, trehalose and glucose, which in turn contributed to conferred drought tolerance (Garg *et al.*, 2002). Galactinol has a positive effect in protecting plants from drought stress and oxidative damage (Taji *et al.*, 2002; Nishizawa *et al.*, 2008). In our study, galactinol showed significant increases after drought treatment, especially in *MLP43* OE plants (Fig. 6). Moreover, overexpressed *GhMLP* led to a two-fold increase in flavonoid contents, which suggested that *MLP* might be involved in flavonoid metaolism (Chen and Dai, 2010). All these results indicated that *MLP43* modulated primary and secondary metabolic profiles, which might be contributed to increased abiotic stress tolerance.

In this study, we dissected the functions of *MLP43* in ABA signals and drought stress responses. First, ABA and drought stress treatments inhibited the expression of *MLP43*, but this inhibition was disturbed in the primary ABA signal components mutants. Second, *MLP43* overexpression enhanced ABA responses in seed germination and improved drought stress tolerance by modulating ABA- and/or drought-responsive gene expression, ROS homeostasis and primary metabolic profiling. Third, *MLP43* promoted RD29A::LUC activity in the reconstituted ABA signalling pathway, and that was dependent upon SnRK2.2/2.3/2.6. Finally, there were direct interactions between MLP43 and SnRK2.6 or ABF1 in the yeast two-hybrid assay. Collectively, we concluded that *MLP43* functioned as a positive regulator upstream of *SnRK2*s, and ABA down-regulated *MLP43* expression as a negative feedback loop, so regulating drought stress responses.

## Supplementary data

Supplementary data is available at *JXB* online.

Supplementary Fig. S1. Phenotypic analysis of another *MLP43* T-DNA insertion line *mlp43-2* (Salk_033347).

Supplementary Fig. S2. Comparison of primary root length and lateral roots number among Col-0, *mlp43-1*, *MLP OE* plants after ABA treatment.

Supplementary Fig. S3. Complementary over-expression of *MLP43* into the *mlp43-1* mutant (*Com-3*) showed drought-tolerance in soil.

Supplementary Fig. S4. Phylogenetic relationships of nine *AtMLP* genes.

Supplementary Fig. S5. Yeast two-hybrid assay to detect the interactions between MLP43/MLP34/MLP168 and the key components in the ABA signalling pathway.

Supplementary Table S1. The primers used for identification of the *mlp43* mutant (Salk_109337 and Salk_033347).

Supplementary Table S2. Detailed information of plasmid construction in this study.

Supplementary Table S3. The primers used for plasmid construction in this study.

Supplementary Table S4. The primers used for real-time quantitative RT-PCR (qRT-PCR) in this study.

Supplementary Table S5. The detailed list of alterations in metabolite profiling under control and drought treatment in Col-0, *mlp43-1* and *MLP OE* plants.

Supplementary Data

## Abbreviations:

ABA: abscisic acid
ABRE: ABA-responsive element
APX: ascorbateperoxidase
CAT: catalase
DAB: 3,3’-diaminobenzidine
EL: electrolyte leakage
GA_3_: gibberellic acid 3
GC-TOF MS: gas chromatograph time-of-flight mass spectrometry
MLP43: major latex protein-like 43
MS: Murashige and Skoog
NBT: nitoblue terazolium chloride
PR10: pathogenesis-related 10
qRT-PCR: real-time quantitative RT-PCR
ROS: reactive oxygen species
SnRK2: SUCROSE NONFERMENTING1-RELATED SUBFAMILY2
SOD: superoxide dismutase
START: star-related lipid-transfer domain.

## References

[CIT0001] Aggelis A John I Karvouni Z Grierson D 1997 Characterization of two cDNA clones for mRNAs expressed during ripening of melon (*Cucumis melo L*) fruits. Plant Molecular Biology 33, 313–322. 9037149 10.1023/a:1005701730598

[CIT0002] Bocian A Zwierzykowski Z Rapacz M Koczyk G Ciesiolka D Kosmala A 2015 Metabolite profiling during cold acclimation of *Lolium perenne* genotypes distinct in the level of frost tolerance. Journal of Applied Genetics . Doi 10. 1007/s13353-015-0293-6. 10.1007/s13353-015-0293-6 26025228

[CIT0003] Chan Z 2012 Expression profiling of ABA pathway transcripts indicates crosstalk between abiotic and biotic stress responses in *Arabidopsis* . Genomics 100, 110–115. 22709556 10.1016/j.ygeno.2012.06.004

[CIT0004] Chen J-Y Dai X-F 2010 Cloning and characterization of the *Gossypium hirutum* major latex protein gene and functional analysis in *Arabidopsis thaliana* . Planta 231, 861–873. 20049612 10.1007/s00425-009-1092-2

[CIT0005] Clough SJ Bent AF 1998 Floral dip: a simplified method for Agro-bacterium-mediated transformation of *Arabidopsis thaliana* . The Plant Journal 16, 735–743. 10069079 10.1046/j.1365-313x.1998.00343.x

[CIT0006] Cutler SR Rodriguez PL Finkelstein RR Abrams SR 2010 Abscisic acid: emergence of a core signaling network. Annual Review of Plant Biology 61, 651–679. 10.1146/annurev-arplant-042809-112122 20192755

[CIT0007] Finkel T 1998 Oxygen radicals and signaling. Current Opinion in Cell Biology 10, 248–253. 9561849 10.1016/s0955-0674(98)80147-6

[CIT0008] Garg AK Kim JK Owens TG Ranwala AP Do Choi Y Kochian LV Wu RJ 2002 Trehalsoe accumulation in rice plants confers high tolerance levels to different abiotic stresses. Proceedings of the National Academy of Science, USA 99, 15898–15903. 10.1073/pnas.252637799 PMC138536 12456878

[CIT0009] Guo D Wong WS Xu WZ Sun FF Qing DJ Li N 2011 *Cis-cinnamic acid-enhanced 1* gene plays a role in regulation of *Arabidopsis* bolting. Plant Molecular Biology 75, 481–495. 21298397 10.1007/s11103-011-9746-4

[CIT0010] Ho T Asada M Kowyama Y Hattori T 1999 ACGT-containing abscisic acid response element (ABRE) and coupling element 3 (CE3) are functionally equivalent. The Plant Journal 19, 679–689. 10571853 10.1046/j.1365-313x.1999.00565.x

[CIT0011] Hu L Li H Pang H Fu J 2012 Responses of antioxidant gene, protein and enzymes to salinity stress in two genotypes of perennial ryegrass (*Lolium perenne*) differing in salt tolerance. Journal of Plant Physiology 169, 149–156. 10.1016/j.jplph.2011.08.020 22088275

[CIT0012] Hubbard KE Nishimura N Hitomi K Getzoff ED Schroeder JI 2010 Early abscisic acid signal transduction mechanisms: newly discovered components and newly emerging questions. Genes & Development 24, 1695–1708. 20713515 10.1101/gad.1953910 PMC2922499

[CIT0013] Inui H Sawada M Goto J Yamazaki K Kodama N Tsuruta H 2013 A major latex-like protein is a key factor in crop contamination by persistent organic pollutants. Plant Physiology 161, 2128–2135. 23404917 10.1104/pp.112.213645 PMC3613481

[CIT0014] Jefferson RA Kavanagh TA Bevan MW 1987 GUS fusions: β-glucuronidase as a sensitive and versatile gene fusion marker in higher plants. The EMBO Journal 20, 3901–3907. 3327686 10.1002/j.1460-2075.1987.tb02730.x PMC553867

[CIT0015] Kim HS Yu Y Snesrud EC Moy LP Linford LD Haas BJ 2005 Transcriptional divergence of the duplicated oxidative stress-responsive genes in the *Arabidopsis* genome. The Plant Journal 41, 212–220. 15634198 10.1111/j.1365-313X.2004.02295.x

[CIT0016] Kim TH Bohmer M Hu H Nishimura N Schroeder JI 2010 Guard cell signal transduction network: advances in understanding abscisic acid, CO_2_, and Ca^2+^ signaling. Annual Review of Plant Biology 61, 561–591. 10.1146/annurev-arplant-042809-112226 PMC3056615 20192751

[CIT0017] Koistinen KM Soininen P Venӓlӓinen TA Hӓyrinen J Laatikainen R Perӓkylӓ M Tervahauta AI Kӓrenlampi SO 2005 Birch PR-10c interacts with several biologically important ligands. Phytochemistry 66, 2524–2533. 16246382 10.1016/j.phytochem.2005.09.007

[CIT0018] Koornneef M Jorna ML Brinkhorst-van der Swan DLC Karssen CM 1982 The isolation of abscisic acid (ABA) deficient mutants by selection of induced revertants in non-germinating gibberellins sensitive lines of *Arabidopsis thaliana* (L.) Heynh. Theoretical and Applied Genetics 61, 385–393. 24270501 10.1007/BF00272861

[CIT0019] Koornneef M Reuling G Karssen CM 1984 The isolation and characterization of abscisic acid-insensitive mutants of *Arabidopsis thaliana* . Physiologia Plantarum 61, 377–383.

[CIT0020] Kwak J Mori IC Pei ZM Leonhardt N Torres MA Dang JL Bloom RE Bodde S Jones JD Schroeder JI 2003 NADPH oxidase *AtrbohD* and *AtrbohF* genes function in ROS-dependent ABA signaling in *Arabidopsis* . The EMBO Journal 22, 2623–2633. 12773379 10.1093/emboj/cdg277 PMC156772

[CIT0021] Lee S Cheng H King KE Wang W He Y Hussain A Lo J Harberd NP Peng J 2002 Gibberellin regulates *Arabidopsis* seed germination via *RGL2*, a GA/RGA-like gene whose expression is up-regulated following imbibitions. Genes & Development 16, 646–658. 11877383 10.1101/gad.969002 PMC155355

[CIT0022] Lee SC Luan S 2012 ABA signal transduction at the crossroad of biotic and abiotic stress responses. Plant, Cell & Environment 35, 53–60. 10.1111/j.1365-3040.2011.02426.x 21923759

[CIT0023] Lisec J Schauer N Kopka J Willmitzer L Fernie AR 2006 Gas chromatography mass spectrometry-based metabolite profiling in plants. Nature Protocol 1, 387–396. 10.1038/nprot.2006.59 17406261

[CIT0024] Lu Y Chen X Wu Y Wang Y He Y Wu Y 2013 Directly transforming PCR-amplified DNA fragment into plant cells is a versatile system that facilitates the transient expression assay. Plos ONE 8, e57171. 23468926 10.1371/journal.pone.0057171 PMC3582559

[CIT0025] Ma Y Szostkiewicz I Korte A Moes D Yang Y Christmann A Grill E 2009 Regulators of PP2C phosphatase activity function as abscisic acid sensors. Science 324, 1064–1068. 19407143 10.1126/science.1172408

[CIT0026] Menkens AE Schindler U Casshmore AR 1995 The G-box: a ubiquitous regulatory DNA elements in plants bound by the CBF family of bZIP proteins. Trends in Biochemistry Science 20, 506–510. 10.1016/s0968-0004(00)89118-5 8571452

[CIT0027] Miller G Suzuki N Ciftci-Yilmaz S Mittler R 2010 Reactive oxygen species homeostasis and signaling during drought and salinity stresses. Plant Cell & Environment 33, 453–467. 10.1111/j.1365-3040.2009.02041.x 19712065

[CIT0027a] Mittler R 2002 Oxidative stress, antioxidants and stress tolerance. Trends in Plant Science 7, 405–410. 10.1016/s1360-1385(02)02312-9 12234732

[CIT0028] Mittler R Vanderauwera S Suzuki N Miller G Tognetti VB Vandepoele K Gollery M Shulaev V Van Breusegem F 2011 ROS signaling: the new wave? Trends in Plant Science 16, 300–309. 21482172 10.1016/j.tplants.2011.03.007

[CIT0029] Mustilli A-C Merlot S Vavasseur A Fenzi F Giraudat J 2002 *Arabidopsis* OST1 protein kinase mediates the regulation of stomatal aperture by abscisic acid and acts upstream of reactive oxygen species production. Plant Cell 14, 3089–3099. 12468729 10.1105/tpc.007906 PMC151204

[CIT0030] Nessler CL Burnett RJ 1992 Organization of the major latex protein gene family in opium poppy. Plant Molecular Biology 20, 749–752. 1450390 10.1007/BF00046460

[CIT0031] Nessler CL Kurz WGW Pelcher LE 1990 Isolation and analysis of the major latex protein genes of opium poppy. Plant Molecular Biology 15, 951–953. 2103486 10.1007/BF00039436

[CIT0032] Nishizawa A Yabuta Y Shigeoka S 2008 Galatinol and raffinose constitute a novel function to protect plants from oxidase damage. Plant Physiology 147, 1251–1263. 18502973 10.1104/pp.108.122465 PMC2442551

[CIT0033] Park SY Fung P Nishimura N Jensen DR Fujii H 2009 Abscisic acid inhibits type 2C protein phosphatases via the PYR/PYL family of START protein. Science 324, 1068–1071. 19407142 10.1126/science.1173041 PMC2827199

[CIT0034] Peleg Z Blumwald E 2011 Hormone balance and abiotic stress tolerance in crop plants. Current Opinion in Plant Biology 14, 290–295. 21377404 10.1016/j.pbi.2011.02.001

[CIT0035] Phee BK Park S Cho JH Jeon JS Bhoo SH Hahn TR 2007 Comparative proteomic analysis of blue light signaling components in the *Arabidopsis* crytochrome 1 mutant. Molecular Cells 23, 154–160. 17464191

[CIT0036] Piskurewicz U Jikumaru Y Kinoshita N Nambara E Kamiya Y Lopez-Molina L 2008 The gibberellic acid singaling repressor RGL2 inhibits *Arabidopsis* seed germination by stimulating abscisic acid synthesis and ABI5 activity. Plant Cell 20, 2729–2745. 18941053 10.1105/tpc.108.061515 PMC2590721

[CIT0037] Radauer C Lackner P Breiteneder H 2008 The Bet v 1 fold: an ancient, versatile scaffold for binding of large, hydrophobic ligands. BMC Evolutionary Biology 8, 286. 18922149 10.1186/1471-2148-8-286 PMC2577659

[CIT0038] Ramel F Sulmon C Bogard M Gouesbet G 2009 Differential patterns of reactive oxygen species and antioxidative mechanisms during atrazine injury and surose-induced tolerance in *Arabidopsis thaliana* plantlets. BMC Plant Biology 9, 28. 19284649 10.1186/1471-2229-9-28 PMC2661893

[CIT0039] Sambrook J Fritsch EF Maniatis T 1989 *Molecular cloning: a laboratory manual* , 2nd edn Cold Spring Harbor: Cold Spring Harbor Labortory Press.

[CIT0040] Shen Q Ho T 1995 Functional dissection of an abscisic acid (ABA)-inducible gene reveals two independent ABA-responsive complexes each containing a G-box and a novel cis-acting element. Plant Cell 7, 295–307. 7734964 10.1105/tpc.7.3.295 PMC160783

[CIT0041] Shi H Ye T Zhong B Liu X Chan Z 2014 Comparative proteomic and metabolomic analyses reveal mechanisms of improved cold stress tolerance in bermudagrass (*Cynodon dactylon* (L). Pers.) by exogenous calcium. Journal of Integrative Plant Biology 56, 1064–1079. 24428341 10.1111/jipb.12167

[CIT0042] Smirnoff N 1993 The roles of active oxygen in the response of plants to water deficit and desiccation. New Phytologist 125, 27–58. 10.1111/j.1469-8137.1993.tb03863.x 33874604

[CIT0043] Taji T Ohsumi C Luchi S Seki M Kasuga M Kobayashi M Yamaguchi-Shinozaki K Shinozaki K 2002 Important roles of drought- and cold- inducible genes for galactinol synthase in stress tolerance in *Arabidopsis thaliana* . The Plant Journal 29, 417–426. 11846875 10.1046/j.0960-7412.2001.01227.x

[CIT0044] Torres MA Dang JL Jones JD 2002 *Arabidopsis* gp91phox homologues AtrbohD and AtrbohF are required for accumulation of reactive oxygen intermediates in the plant defense response. Proceedings of the National Academy of Science, USA 8, 517–522. 10.1073/pnas.012452499 PMC117592 11756663

[CIT0045] Wang Y Li L Ye T Lu Y Chen X Wu Y 2013 The inhibitory effect of ABA on floral transition is mediated by ABI5 in *Arabidopsis* . Journal of Experimental Botany 64, 675–684. 23307919 10.1093/jxb/ers361 PMC3542054

[CIT0046] Wang Y Yang L Zheng Z Grumet R Loescher W Zhu J-K Yang P Hu Y Chan Z 2013 Transcriptomic and physiological variations of three *Arabidopsis* ecotypes in response to salt stress. Plos ONE . 8, e69036. 23894403 10.1371/journal.pone.0069036 PMC3720874

[CIT0047] Winter D Vinegar B Nahal H Ammar R Wilson GV Provart NJ 2007 An ‘electronic fluorescent pictograph’ browser for exploring and analyzing large-scale biological data sets. PloS ONE 2, e718. 17684564 10.1371/journal.pone.0000718 PMC1934936

[CIT0048] Wu FZ Lu TC Shen Z Wang BC Wang HX 2008 N-terminal acetylation of two major latex proteins from *Arabidopsis thaliana* using electrospray ionization tandem mass spectrometry. Plant Molecular Biology Reporter 26, 88–97.

[CIT0049] Yoo SD Cho YH Sheen J 2007 *Arabidopsis* mesophyll protoplasts: a versatile cell system for transient gene expression analysis. Natural Protocol 2, 1565–1572. 10.1038/nprot.2007.199 17585298

