# Supplementary material for: Major latex protein-like protein 43 (MLP43) functions as a positive regulator during abscisic acid responses and confers drought tolerance in Arabidopsis thaliana
Source: J Exp Bot. 2015 Oct 27;67(1):421–34. doi: 10.1093/jxb/erv477 (PMC4682443; doi:10.1093/jxb/erv477)
Supplement: Supplementary Data [file supp_67_1_421__index.html]

 Major latex protein-like protein 43 (MLP43) functions as a positive regulator during abscisic acid responses and confers drought tolerance in Arabidopsis thaliana — Supplementary Data 

# *Major latex protein-like protein 43* (*MLP43*) functions as a positive regulator during abscisic acid responses and confers drought tolerance in *Arabidopsis thaliana*

## Supplementary Data

Data files

- Supplementary\_Figures\_legends.docx - Supplementary Data
- Supplementary\_Fig.1.tif - Supplementary Data
- Supplementary\_Fig.2.tif - Supplementary Data
- Supplementary\_Fig.3.tif - Supplementary Data
- Supplementary\_Fig.4.tif - Supplementary Data
- Supplementary\_Fig.5.tif - Supplementary Data
- Supplementary\_tables\_S1\_S5.pdf - Supplementary Data
