# Supplementary material for: Major latex protein-like protein 43 (MLP43) functions as a positive regulator during abscisic acid responses and confers drought tolerance in Arabidopsis thaliana
Source: J Exp Bot. 2015 Oct 27;67(1):421–34. doi: 10.1093/jxb/erv477 (PMC4682443; doi:10.1093/jxb/erv477)
Supplement: Supplementary Data [file supp_erv477_Supplementary_Figures_legends.docx]

Supplementary Fig. S1. Phenotypic analysis of another *MLP43* T-DNA insertion line *mlp43-2* (Salk_033347). (A) Confirmation of the transgenic plants with *MLP43* overexpression by western-blot. Anti-GFP antibody was used to detect the expression levels of MLP43-GFP fusion protein in wildtype (Col-0) and *MLP43* overexpressed transgenic, respectively. The whole cell lysate was used as loading control. (B) ABA responses analysis of *mlp43-2* plants in seed germination assay. Photos were taken to document the ABA insensitivity of *mlp43-2* plants at the 5^th^ day after stratification. (C) Relative expression levels of *MLP43* in wildtype (Col-0) and Salk_033347 lines. The data represent means + SE of two replicates. (D) Germination rates of green cotyledons in 1.0 μM ABA plates at the 5^th^ day after stratification. Data represent the means + SEs of three replicated experiments (n>30 for each experiment).

Supplementary Fig. S2. Comparison of primary root length and lateral roots number among Col-0, *mlp43*, and *MLP43 OE* plants after ABA treatment. The data represent means + SEs of three reproducible experiments (n=15). 4-day-old plants on MS plates were transformed onto fresh MS plates supplemented with indicated ABA, and the primary root length was calculated at the 7^th^ day after transferring.

(A) The primary root length comparison under indicated ABA treatment. (B) The lateral root numbers comparison under indicated ABA treatment.

Supplementary Fig. S3. Complementary expression of *MLP43* into *mlp43-1* (*Com-3*) rescued its drought-sensitivity in soil.

(A) The plants were well watered for 14 days and then withholding water for 21 days. Photos were taken after 2 days with re-watering. (B) The survival rates were calculated based on the results in (A). The data represent means + SEs of three reproducible experiments, and for each genotype (n=27, *p*<0.01).

Supplementary Fig. S4. Phylogenetic relationships of *AtMLP* genes. The unrooted phylogenetic tree was created in Vector NTI software based on nine full-length amino acid sequences of MLP alleles in *Arabidopsis*.

Supplementary Fig. S5. Yeast two hybrid assay (Y2H) to detect the interactions between MLP43/MLP34/MLP168 and the key components in ABA signaling pathway. (A) MLP168 interacts with wildtype ABI5 in Y2H assay but not with the mutated proteins carrying mutations of phosphoamino acids (S42A, S145A and T201A) in ABI5. (B) MLP43 interacts with SnRK2.6 and ABFs in Y2H assay
