# Supplementary figures and images for: Major latex protein-like protein 43 (MLP43) functions as a positive regulator during abscisic acid responses and confers drought tolerance in Arabidopsis thaliana
Source: J Exp Bot. 2015 Oct 27;67(1):421–34. doi: 10.1093/jxb/erv477 (PMC4682443; doi:10.1093/jxb/erv477)

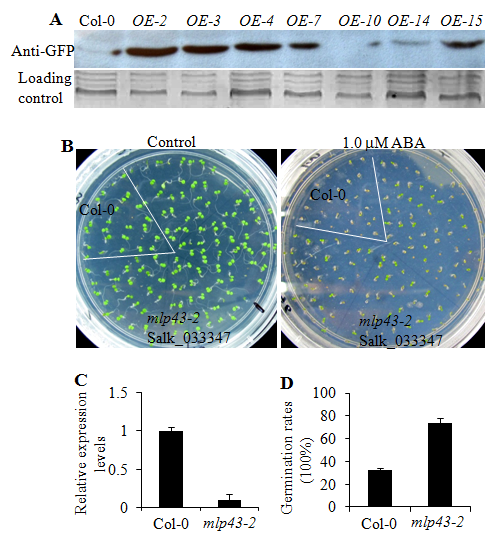

Supplement: Supplementary Data [file supp_erv477_Supplementary_Fig.1.tif]

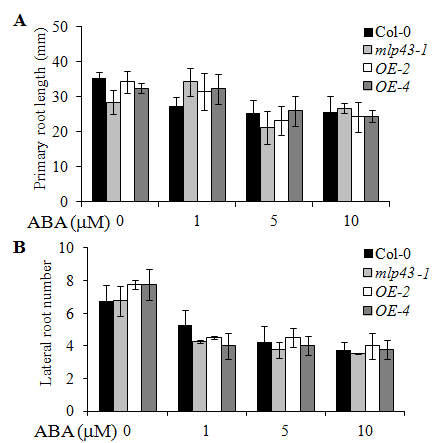

Supplement: Supplementary Data [file supp_erv477_Supplementary_Fig.2.tif]

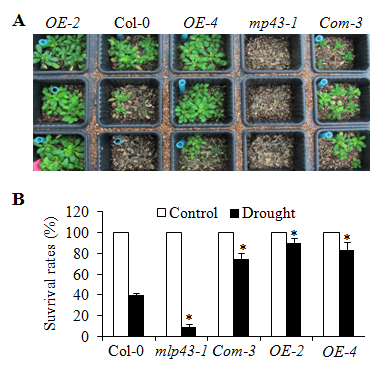

Supplement: Supplementary Data [file supp_erv477_Supplementary_Fig.3.tif]

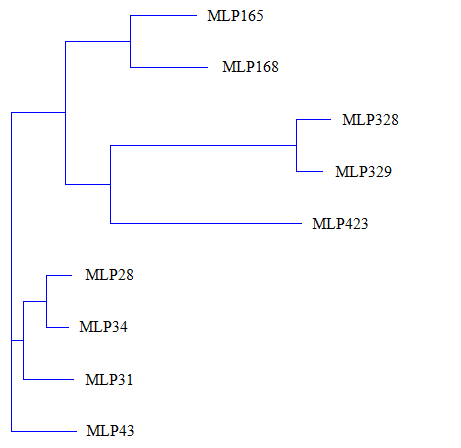

Supplement: Supplementary Data [file supp_erv477_Supplementary_Fig.4.tif]

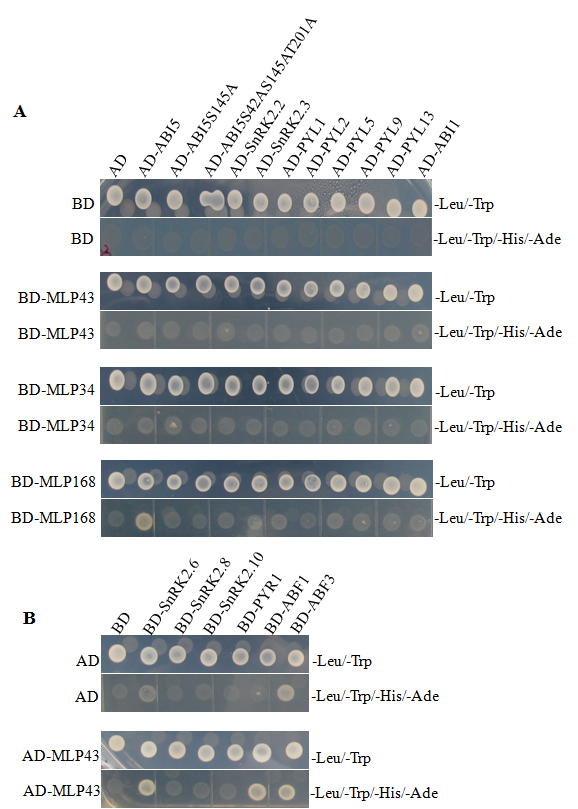

Supplement: Supplementary Data [file supp_erv477_Supplementary_Fig.5.tif]
