# Supplementary material for: Major latex protein-like protein 43 (MLP43) functions as a positive regulator during abscisic acid responses and confers drought tolerance in Arabidopsis thaliana
Source: J Exp Bot. 2015 Oct 27;67(1):421–34. doi: 10.1093/jxb/erv477 (PMC4682443; doi:10.1093/jxb/erv477)
Supplement: Supplementary Data [file supp_erv477_Supplementary_tables_S1_S5.pdf]

Supplementary Tables

Table S1

Table S1. The primers used for identification of *mlp43* mutant (Salk_109337 and Salk_033347).

LBb1.3	5’-ATTTTGCCGATTTCGGAAC-3’
109337-RP	5’-ACTTTCCATCTGCACCATTTG-3’
109337-LP	5’-ACATCACGGTCGATCTTAACG-3’
033347-RP	5’-TTGGAAAAGTAAGCAATGGTCTC-3’
033347-LP	5’-CTTGGAGTGTAAATGCGAAGC-3’

Table S2

Table S2. The detail information of plasmids construction in this study.

**Plasmids Names**	**Cloning Sites**	**Vectors**	**References**
Pro35S::MLP43-GFP	XhoI/KpnI	pGFP	
pBA-MLP43-GFP	XhoI/SacI	pBA	
ProMLP43::GUS	XbaI/XmaI	pBI101.1	
AD-MLP43	EcoRI/SacI	pGADT	Clontech
BD-MLP43	EcoRI/PstI	pGBKT	Clontech
BD-MLP34	NcoI/EcoRI	pGBKT	Clontech
BD-MLP168	NcoI/EcoRI	pGBKT	Clontech
AD-PYL1	ClaI/SacI	pGADT	Clontech
AD-PYL2	EcoRI/ClaI	pGADT	Clontech
AD-PYL5	EcoRI/ClaI	pGADT	Clontech
AD-PYL9	EcoRI/ClaI	pGADT	Clontech
AD-PYL13	ClaI/SacI	pGADT	Clontech
AD-ABI1	BamHI/XhoI	pGADT	Clontech
AD-ABI5	EcoRI/XhoI	pGADT	Clontech
AD-ABI5S42A	EcoRI/XhoI	pGADT	Clontech
AD-ABI5S42AS145AT201A	EcoRI/XhoI	pGADT	Clontech
AD-SnRK2.2	EcoRI/XhoI	pGADT	Clontech
AD-SnRK2.3	EcoRI/XhoI	pGADT	Clontech
BD-SnRK2.6	NcoI/EcoRI	pGBKT	Clontech
BD-SnRK2.8	EcoRI/XmaI	pGBKT	Clontech
BD-SnRK2.10	EcoRI/XmaI	pGBKT	Clontech
BD-PYR1	EcoRI/PstI	pGBKT	Clontech
BD-ABF1	BamHI/SalI	pGBKT	Clontech
BD-ABF3	BamHI/SalI	pGBKT	Clontech

Table S3

Table S3. The primers used for plasmids construction.

Pro35S::MLP43-GFP	F: 5’-GGCTCGAG ATGGCAGAAGCGTCTAGTTTGGTGG-3’
	R: 5’CCG GGTACCCTCTTCCTCGGCCAAGAGATGTTCG-3’
pBA-MLP43-GFP	F: 5’-CCGCTCGAGATGGCAGAAGCGTCTAGTTTGGTGG-3’
	R: 5’-CGAGCTCCTATTCCTCGGCCAAGAGATGTTCG-3’
ProMLP43::GUS	F: 5’-GCTCTAGACTCACAAAACCTTGTTCCGGAGG-3’
	F: 5’-TCCCCCGGGTCGGTAAACATGTGATGGAA-3’
MLP43-AD/BD	F: 5’-GGAATTCATGGCAGAAGCGTCTAGTTT-3’
MLP4343-AD	R: 5’-CGAGCTCCTATTCCTCGGCCAAGAGA-3’
MLP43-BD	R: 5’-AACTGCAGCTATTCCTCGGCCAAGAGA-3’
MLP168-BD	F: 5’-CATGCCATGGAGATGGTAGAGGCAGAGGTTGA-3’
	R: 5’-GGGAATTCCTAACCCTCAGACAATAG-3’
MLP34-BD	F: 5’-CATGCCATGGAG ATGGCAAAAACTGAAGCT-3’
	R: 5’-GGGAATTCCTATTCCTCGGCCAA-3’
PYL1-AD	F: 5’-CCATCGATACATGGCGAATTCAGAGTCCTC-3’
	R: 5’-CGAGCTCTTACCTAACCTGAGAAGAGTT-3’
PYL2-AD	F: 5’-GGGAATTCATGAGCTCATCCCCGGC-3’
	R: 5’-CCATCGATTTATTCATCATCATGCATAG-3’
PYL5-AD	F: 5’-GGGAATTCATGAGGTCACCGGTGCAA-3’
	R: 5’-CCATCGATTTATTGCCGGTTGGTACT-3’
PYL9-AD	F:5’-GGGAATTC ATGATGGACG GCGTTGA-3’
	R: 5’-CCATCGATTCACTGAGTAATGTCCTG-3’
PYL13-AD	F: 5’-CCATCGATACATGGAAAGTTCTAAGCAAAA-3’
	R: 5’-CGAGCTCTTACTTCATCATTTTCTTTG-3’
ABI5-AD	F: 5’-GGAATTCATGGTAACTAGAGAAACGAA-3’
	R: 5’-CCGCTCGAGTTAGAGTGGACAACTCGGGTT-3’
ABI1-AD	F: 5’-CGGGATCCGTATGGAGGAAGTATCTCCGGC-3’
	R: 5’-GGCTCGAGTCAGTTCAAGGGTTTGCTCTT-3’
ABF1-BD	F: 5’-CGGGATCCCGATGGGTACTCACATTGATATC-3’
	R: 5’-GCGTCGACTCACCTTCTTACCACGGACCG-3’
ABF3-BD	F: 5’-CGGGATCCCGATGGGGTCTAGATTAAACTTC-3’
	R: 5’-GCGTCGACTGGCTGGCGCAGAGGCTCCAGA-3’
SnRK2.2-AD	F: 5’-GGAATTCATGGATCCGGCGACTAATTC-3’
	R: 5’-CCGCTCGAGTCAGAGAGCATAAACTATCT-3’
SnRK2.3-AD	F: 5’-GGAATTCATGTATCGAGCTCCGGTGAC-3’
	R: 5’-CCGCTCGAGTTAGAGAGCGTAAACTATCTC-3’
SnRK2.6-BD	F: 5’- CATGCCATGGATGGATCGACCAGCAGTGA-3’
	R: 5’-GGGAATTCTCACATTGCGTACACAAT-3’
SnRK2.8-BD	F: 5’-GGAATTCATGGAGAGGTACGAAATAGTGAA-3’
	R: 5’-TCCCCCGGGTCACAAAGGGGAAAGGAGATCA-3’
SnRK2.10-BD	F: 5’-GGAATTCATGGACAAGTACGAGCTTGTTA-3’
	R: 5’-TCCCCCGGGTTAACTGACTCGGACTTCTCCCA-3’
PYR1-BD	F: 5’-GGAATTCATGCCTTCGGAGTTAACACC-3’

R: 5’-AACTGCAGTCACGTCACCTGAGAACCAC-3’

Table S4

Table S4. The primers used for real-time quantitative RT-PCR.

MLP43	F: 5’-GGAAATACGTTCATGATGGAAA-3’
	R: 5’-TTGGAGTGTAAATGCGAAGC-3’
ABA1	F: 5’- GACTGGGTCCTTGGAGGTAA-3’
	R: 5’- CATCGGCTTTGTCAGTGAGT-3’
ABA2	F: 5’- TCCAAGCATGCTGTTCTAGG-3’
	R: 5’- AAATGAGCCAAAGCGAGTTT-3’
ABI1	F: 5’-AGATGGTCGGTTTGATCCTC-3’
	R: 5’-AGTTCGCTACCTGAGAACCG-3’
ABI2	F: 5’-TCAAGATCCATTGGCGATAG-3’
	R: 5’-CAAATCGCACACTTCTTCGT-3’
DREB2A	F: 5’-AACCTGTCAGCAACAACAGC-3’
	R: 5’-AAACACATCGTCGCCATTTA-3’
HAB1	F: 5’-TCTAGGTCCATCGGTGACAG-3’
	R: 5’-TTTCGCAGACTTCTTGGTTG-3’
KIN1	F: 5’-GCAATGTTCTGCTGGACAAG-3’
	R: 5’-TACACTCTTTCCCGCCTGTT-3’
NCED3	F: 5’ -TTGATGCTCCAGATTGCTTC-3’
	R: 5’- GGACCCTATCACGACGACTT-3’
RAB18	F: 5’-AGCTCTAGCTCGGAGGATGA-3’
	R: 5’-CATGATGACCTGGCAACTTC-3’
RbohD	F: 5’- AACAACAGGTGGCTGTTTACC-3’
	R: 5’-TGTGATTGAGAAAGGATGCC-3’
RbohF	F: 5’-TTCAGTATCCGTGGGCAATA-3’
	R: 5’-CACTCCTGCGAAAGATCAAA-3’
RD20	F: 5’-GGATTTCGTGACCTTGGTTT-3’
	R: 5’-TAAACCGGCAATAATGGTGA-3’
RD29A	F: 5’-AGGAACCACCACTCAACACA-3’
	R: 5’-GCTCATGCTCATTGCTTTGT-3’
RD29B	F: 5’-ACGAGCAAGACCCAGAAGTT-3’
	R: 5’-AGGAACAATCTCCTCCGATG-3’
β-ACTIN8	F: 5’-AGTGGTCGTACAACCGGTATTGT-3’
	R: 5’-GAGGATAGCATGTGGAAGTGAGAA-3’

Table S5

Table S5. The detailed list of relative folds alterations with metabolite profiling under control and drought treatment in Col-0, mlp43 and MLP OE plants. ND: not detected; the values in this table indicate folds changes.

Metabolites	*mlp43*-mock vs. Col-mock	*OE-2*-mock vs. Col- mock	*mlp43*-Dr. vs. Col-Dr.	*OE-2*-Dr. vs. Col- Dr.
D-(+)-turanose	1.2928	-0.6211	-1.8467	3.4855
D-(+)-trehalose	1.0295	0.2802	-1.8477	6.3784
Maltose	0.8364	0.1456	-5.9036	1.3625
D-(+)-galactose	ND	0.2671	-0.6023	-1.6630
D-(+)-cellobiose	-0.8911	1.5569	-0.7989	1.8883
D-psicose	1.0401	-1.4912	-0.1577	1.1133
L-(-)-sorbose	-0.4544	-2.0302	-1.7593	1.0508
D-galactose	0.9054	-0.3398	-3.7509	0.3531
D-glucose	-0.9717	-0.1240	-3.0409	5.1033
Citrulline	0.2431	0.8408	-2.8620	1.5951
L-glutamine	1.1987	0.1261	0.6324	0.0365
L-proline	0.2502	1.0781	-1.6644	6.4170
Glycine	0.7501	-1.4510	ND	-1.5561
L-valine	2.0051	-0.9602	-0.0952	2.6086
Serine	0.8712	1.0707	-0.3531	1.7107
L-threonine	-1.1230	-1.3525	-4.6917	1.1523
L-lysine	-0.3012	-1.0067	-1.4789	2.5674
L-alanine	0.3577	-0.3353	-0.0365	11.7320
Octadecanoic acid	-0.6841	-0.2986	-1.7069	8.6853
Phosphoric acid	-0.5602	-1.1020	0.7954	6.9419
Cinnamic acid	-1.6764	-1.1612	-2.0426	-1.0335
Hexadecanoic acid	0.2310	0.1310	2.8620	4.1404
2-ketoglutaric acid	0.4697	0.1804	-0.6323	1.2358
Glutamic acid	0.9325	-2.9988	1.0196	-1.6766
2-butenedioic acid	1.0971	1.510	-5.7361	-0.5126
Propanoic acid	-0.0897	-0.5095	-0.6048	-1.3319
Ethanimidic acid	1.7649	-0.3303	-1.2312	2.6713
Galactinol	0.1409	-0.3133	1.8476	2.4783
α-D-galactopyranoside	-0.1335	-0.0031	0.6023	-1.9018
Myo-Inositol	-0.0341	-0.2187	1.7593	2.1501
Androst-2-en-17-amine	0.2022	-0.3458	3.1459	3.2062
Glycerol	-1.8229	-0.6387	-0.0983	0.8192
Borane	-0.3663	0.2110	0.3617	-1.6835
Ethylene glycol	1.1731	0.8680	0.5168	1.6064
